# Method for measuring tri-axial lumbar motion angles using wearable sheet stretch sensors

**DOI:** 10.1371/journal.pone.0183651

**Published:** 2017-10-11

**Authors:** Akio Yamamoto, Hiroyuki Nakamoto, Tokiya Yamaji, Hideo Ootaka, Yusuke Bessho, Ryo Nakamura, Rei Ono

**Affiliations:** 1 Department of Community Health Science, Graduate School of Health Science, Kobe University, Kobe, Hyogo, Japan; 2 Department of System Science, Graduate School of System Informatics, Kobe University, Kobe, Hyogo, Japan; 3 Bando Chemical Industries, Ltd., Kobe, Hyogo, Japan; Northwestern University, UNITED STATES

## Abstract

**Background:**

Body movements, such as trunk flexion and rotation, are risk factors for low back pain in occupational settings, especially in healthcare workers. Wearable motion capture systems are potentially useful to monitor lower back movement in healthcare workers to help avoid the risk factors. In this study, we propose a novel system using sheet stretch sensors and investigate the system validity for estimating lower back movement.

**Methods:**

Six volunteers (female:male = 1:1, mean age: 24.8 ± 4.0 years, height 166.7 ± 5.6 cm, weight 56.3 ± 7.6 kg) participated in test protocols that involved executing seven types of movements. The movements were three uniaxial trunk movements (i.e., trunk flexion-extension, trunk side-bending, and trunk rotation) and four multiaxial trunk movements (i.e., flexion + rotation, flexion + side-bending, side-bending + rotation, and moving around the cranial–caudal axis). Each trial lasted for approximately 30 s. Four stretch sensors were attached to each participant’s lower back. The lumbar motion angles were estimated using simple linear regression analysis based on the stretch sensor outputs and compared with those obtained by the optical motion capture system.

**Results:**

The estimated lumbar motion angles showed a good correlation with the actual angles, with correlation values of r = 0.68 (SD = 0.35), r = 0.60 (SD = 0.19), and r = 0.72 (SD = 0.18) for the flexion-extension, side bending, and rotation movements, respectively (all *P* < 0.05). The estimation errors in all three directions were less than 3°.

**Conclusion:**

The stretch sensors mounted on the back provided reasonable estimates of the lumbar motion angles. The novel motion capture system provided three directional angles without capture space limits. The wearable system possessed great potential to monitor the lower back movement in healthcare workers and helping prevent low back pain.

## Introduction

Low back pain (LBP) is a disabling complaint experienced by approximately two-thirds of adults at some point in their lives and requires expensive treatment [1,2]. Studies showed a relatively high prevalence of LBP in nurses and healthcare workers, relative to workers in other industries [3–6]. LBP episodes require primary care consultation, regardless of the pain intensity, because of the subjective nature of LBP [7]. Furthermore, chronic LBP leads to absenteeism and presenteeism, which are serious factors affecting productivity and quality of work [8,9]. The number of workers in the healthcare industry has been increasing with the ongoing aging of society. Hence, the issue of LBP prevention is of utmost importance.

Epidemiological studies revealed that physical movements, including trunk flexion and rotation, are the physical risk factors associated with the occurrence of LBP in occupational settings [10–13]. This is partly because larger movement angles increase the load on the lower back by increasing the torque, which causes both increased disc compression and shear forces [14]. Cumulative moderate back load and large peak loads are independently associated with LBP occurrence [15]. Based on an assessment of their posture and movements during work time, workers need to change their working behavior to reduce the loads they impose on their lower back. However, an accurate assessment of the physical movements in occupational settings based on a reliable monitoring system is often difficult to achieve.

Using questionnaire surveys is one of the most popular methodologies for assessing physical movements during working time, and often yield results with low reliability and validity [16] because subjects often overestimate their physical movements [17]. The direct observation method provides a rough picture of the time spent in certain postures [16], but it is only suitable for monitoring a limited working area. The video recording method was adapted to measure the trunk angles in an automobile factory, where workers frequently bend forward and are forced to assume an unnatural posture [13]. However, the capture area was limited to that in the cameras’ field of view. Healthcare workers usually walk around extensively within their institutions to take care of many patients. The capture space is too small to entirely record their movements. Therefore, a new motion capture system without spatial limitations is required to measure the movements of these workers.

Recent progress in micro-electromechanical systems enabled inertial measurement unit (IMU) sensors to be used as wearable human motion capture system for gait analysis and trunk and pelvic kinematics [18–21]. IMU sensors include tri-axial accelerometers and gyroscopes, and sometimes magnetometers, in a small and light unit. The advantages of the IMU sensors are their small size and robustness compared to optical motion capture systems. However, the disadvantages of the IMU sensors involve computational problems in estimating the angles.

Advancements in material engineering technology have resulted in the production of wearable sensors that track human movements. One such example is the sheet stretch sensor [22], which is a flexible, thin sensor designed for mounting on curved surfaces. This sensor is capable of stretching along with the skin without interfering with the wearer’s body movements. A previous study proposed the idea of angle measurement with a stretch sensor using simple linear regression [23]. By using a stretch sensor, we may monitor the lower back movements, namely flexion, side bending, and rotation, of healthcare workers during their work hours. Therefore, this study aims to investigate the validity of the lumbar motion measurements obtained from stretch sensors.

## Materials and methods

### Participants

A group of six healthy young adults (i.e., three males and three females) participated in the study (mean age: 24.8 ± 4.0 years, height 166.7 ± 5.6 cm, weight 56.3 ± 7.6 kg). We obtained informed consent from all the participants before including them in the experiment. This study was approved by the ethical committee of the Kobe University Graduate School of Health Science in accordance with the Helsinki declaration.

### Experimental procedures and measurements

The participants were asked to stand on a flat floor in an upright position with their feet shoulder-width apart and their arms close to their body. They performed seven types of lumbar movements. The movements consisted of three uniaxial trunk movements (i.e., trunk flexion, trunk side-bending, and trunk rotation) and four multiaxial trunk movements (i.e., flexion + rotation, flexion + side-bending, side-bending + rotation, and moving around cranial–caudal axis). The movement conditions are listed in the second left column of Table 1. Each trial lasted for 30 s. The participants were instructed to hold their necks naturally, while trying to keep them stable. They were also asked to look straight ahead during the test. The movement frequency was once in eight seconds (= 0.125 Hz). The movement speed was adjusted to match the rhythm of an electronic metronome beating at 30 times/min.

**Table 1:** ROM values and RMS of errors between actual and estimated lumbar angles.

Trial	Movement Condition	ROM (degree)						Error (degree)					
		Flexion-extension		Side Bending		Rotation		Flexion-extension		Side Bending		Rotation	
		mean	SD	mean	SD	mean	SD	mean	SD	mean	SD	mean	SD
1	Flexion	33.82	7.34	7.06	1.88	5.36	2.81	2.36	1.15	1.21	0.25	0.73	0.38
2	Side-bending	7.76	2.92	12.42	6.17	7.79	2.55	1.54	0.65	1.50	0.13	0.74	0.28
3	Rotation	6.30	2.63	11.48	4.49	11.22	4.69	1.98	1.41	2.06	0.88	0.93	0.46
4	Flexion + Rotation	24.07	5.97	18.64	5.77	10.83	5.23	1.85	0.59	2.07	0.89	1.12	0.72
5	Rotation + Side-bending	10.79	5.53	18.01	5.83	12.46	4.85	2.08	0.84	2.16	0.48	2.16	1.41
6	Side-bending + Flexion	24.47	7.00	17.26	6.73	9.41	2.77	2.75	0.89	2.17	0.63	1.31	0.70
7	Moving around cranial-caudal axis	31.19	11.73	16.86	6.00	10.26	1.84	3.91	3.08	2.71	0.82	1.46	0.52
Over all		19.77	11.36	14.53	4.31	9.62	2.38	2.35	0.79	1.98	0.49	1.21	0.50

ROM: range of motion, SD: standard deviation

### Apparatus

Four flexible stretch sensors (C-STRETCH®, Bando Chemical Industries, Ltd., Japan) were attached to each participant’s back using adhesive jelly. The sensor system was composed of a sensor, a transmitter, and input and output cables. The size of the transmitter was W51D34H25 mm and the weight of the receiver was 23.5 g. The size of the stretchable sensor was W10L50 (mm) in the form of a sheet. The stretch sensor is extremely thin (150 μm), flexible with a wide dynamic range (up to 100% with 0.8 MPa of stretching stress), and light (1.1 g/cm^3^). The sensor is composed of three elastomer layers and two electrode layers. The parallel plate structure works as a capacitator. Since the elastomer sheets were almost incompressible, the capacitance of the stretch sensor is proportional to its areal strain. In fact, the capacitance of the sensor showed high linearity to the square of the area of the sensing part (R^2^ > 0.98) [22]. The measurement error in length of the sensing part was reported to be less than 1.5 mm in 40 and 60 mm of contraction [24]. Moreover, the capacitator type of stretch sensor shows high repeatability of measurements [22].

**Fig 1:**
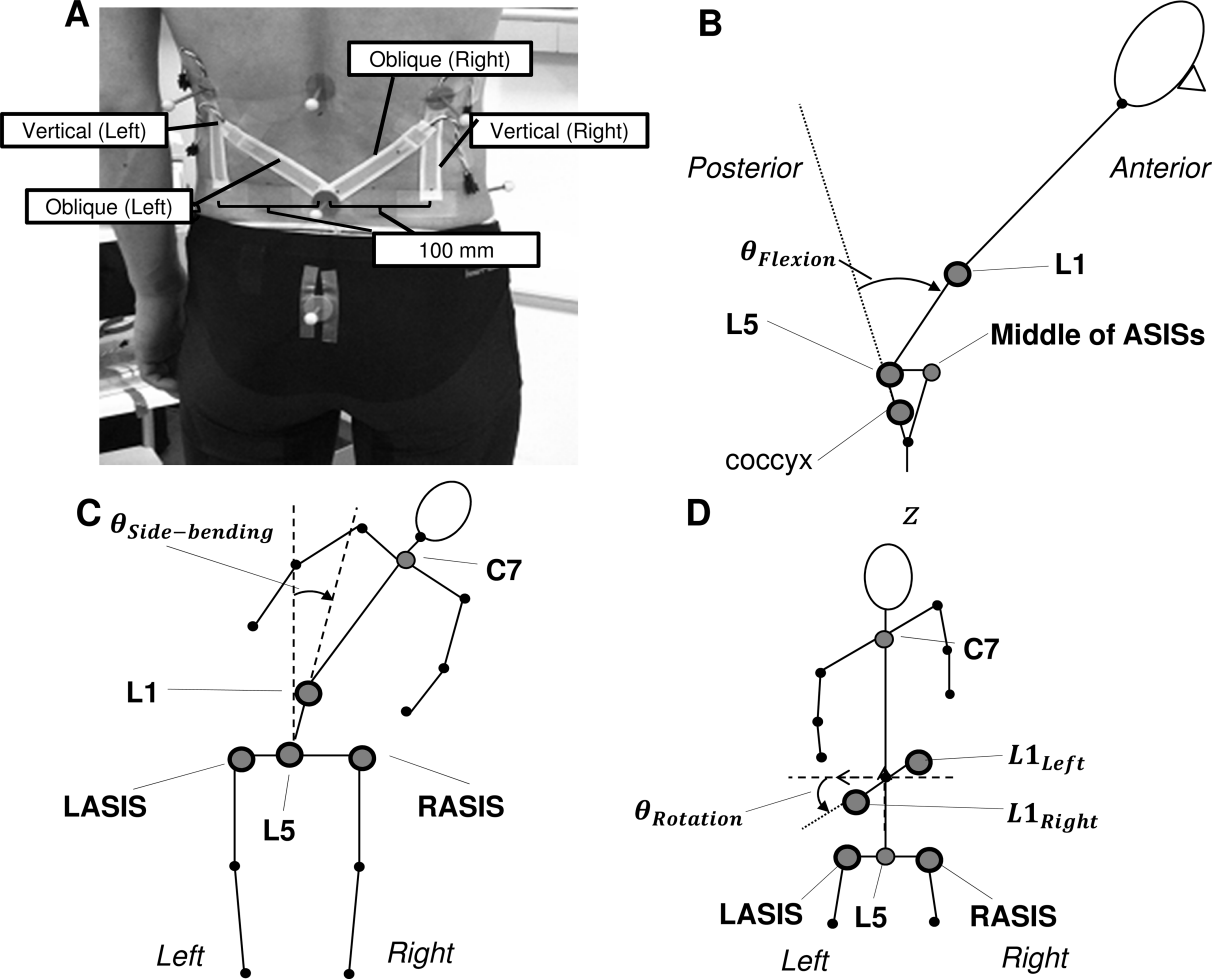
Setup of the experiment and definitions of the three lumbar joint angles. (A) Four sheet stretch sensors and reflective markers attached to a participant’s back. The lower ends of the sensor are at the L5 level. Two sensors are vertically attached, while the other two are attached in a direction diagonal to the L5 level. Reflective markers are attached to ten anatomical landmarks. (B–D) Stick diagram showing the definitions of the lower-back motion angles for flexion-extension (B), side-bending (C), and rotation (D). The gray circles represent the spherical markers placed on the anatomical landmarks. The markers with a bold black edge line are used for the angle calculation in each direction.

Fig 1A shows the four stretch sensors attached to a subject’s back. Two vertical sensors were placed 10 cm from the midpoint of the participant’s back at the L5 level. The two other sensors were attached obliquely from the L5 level to the middle of the subject’s back at a 45° angle. The initial length of all the sensors was 7 cm. The pre-strain was aimed at measuring contraction of the sensor. This helped to measure backward bending movements (extension).

An optical motion capture system (Optitrack, NaturalPoint, USA) consisting of eight cameras arranged in a circle around the participants was used to record the motion of ten reflective markers placed on each participant’s anatomical landmarks. The reflective markers measuring 11 mm in diameter were fixed with a double-sided adhesive tape at the following locations: C7, right and left acromions; three markers at the 1st lumber spine level (L1_left_, L1, and L1_right_); right and left anterior superior iliac spine (ASIS); 5th lumber spine; and the coccyx.

The optical motion tracking system generated a starting trigger signal at the same time to record the marker data using the software (Motive Tracker ver.1.9.0. NaturalPoint. USA). The trigger signal started a 16-bit data acquisition system (PowerLab 16/35, ADInstruments, New Zealand) to record all stretch sensor signals at a sampling frequency of 100 Hz without any time delay. Both the marker and sensor data were stored on a hard disk for off-line analysis.

### Data analysis

All the stretch sensor signals were offset by the first frame data of each trial to obtain the displacement values. Both the marker and stretch sensor data were then low-pass filtered using a fourth-order Butterworth filter with a 10 Hz cutoff frequency. The cutoff frequency was determined based on the movement frequency of the experiment, 0.125 Hz, as well as power spectrum analysis. The marker data were used to calculate the three-dimensional angles relative to the pelvis coordinate system using a Cardan X–Y–Z (flexion–lateral bending–rotation) rotation sequence. Fig 1 defines the three lumber motion angles: Fig 1B, 1C and 1D relate to flexion-extension, side bending, and rotation, respectively.

We estimated the three lumbar motion angles using the signals obtained from the stretch sensors attached to each participant’s back. The stretch sensor measured the sensor length displacement. The sensors stretched together with the skin underneath the sensor. Moreover, the output signals were proportional to the stretch displacement of the skin. Thus, a joint angle can be obtained by a simple linear regression using the sensor outputs [23].

For the lumbar flexion angle, we used the average length of two vertical sensors (Δ*l*_1_ and Δ*l*_2_) to estimate the flexion angle.

$$\Delta \theta_{FX}^{EST}=\alpha_{1}(\Delta l_{1}+\Delta l_{2})/2$$ (1)

Where $\Delta \theta_{FX}^{EST}$ represents estimated angle in the lumbar flexion-extension direction, and α_1_ represents regression coefficient obtained by a simple regression analysis in the lumbar flexion-extension movements.

For the lumbar side-bending angle, the difference between the two vertical sensors was calculated, and the difference data were used to estimate the side-bending angle using the following equation:

$${∆\theta }_{SB}^{EST}=\alpha_{2}(∆l_{1}-∆l_{2})$$ (2)

Where ${∆\theta }_{SB}^{EST}$ represents the estimated angle in the lumbar side-bending direction, and α_2_ represents the regression coefficient obtained by a simple regression analysis in the lumbar side-bending movements.

For the lumbar rotation angle, all four sensor outputs were used for estimation. The stretch values obtained from the vertical sensors were subtracted from those obtained from the oblique sensors to reduce the cross-talk effect and obtain the adjusted oblique outputs (Δ*l*‘_3_ and Δ*l*‘_4_). The difference between the adjusted oblique outputs was then calculated and used to estimate the rotation angle.

$$\theta_{RT}^{ACT}=\alpha_{3}(∆{\overset{´}{l}}_{3}-∆{\overset{´}{l}}_{4})$$ (3)

Where ${∆\theta }_{RT}^{EST}$ represents estimated angle in the lumbar rotation, and α_*3*_ represents regression coefficient obtained by a simple regression analysis in the lumbar rotation movements. All the regression coefficients (α_*1*_, α_*2*_, and α_*3*_) were obtained based on the first 10 s of each datum.

### Statistics

The range of motion (ROM) in the flexion-extension, side-bending, and rotation angle were calculated for both actual and estimated angles. The accuracy of the angle estimation algorithm was evaluated using the root–mean–square (RMS) of the residual error and the Pearson’s correlation coefficient between the actual and estimated angles. Agreements between ROM-measurements were visualized by the plot of the difference between each paired measurement against the mean value of both (Bland-Altman plot) [25]. The plot also shows a dashed line at ±1.96 standard deviation (SD); thus, 95% of the measurements are within these two bounds. The statistical significance level was set to *P* < 0.05. The data were presented using the expression mean ± SD. The data were then analyzed using MATLAB® R2015b (MathWorks, USA).

## Results

Table 1 lists the ROM in all seven trials as obtained by the optical motion capture system. The average ROM values were 19.77° (SD = 11.36) in flexion, 14.53° (SD = 4.31) in side-bending, and 9.62° (SD = 2.38) in rotation. The ROM values varied depending on the movement conducted in each trial.

**Fig 2:**
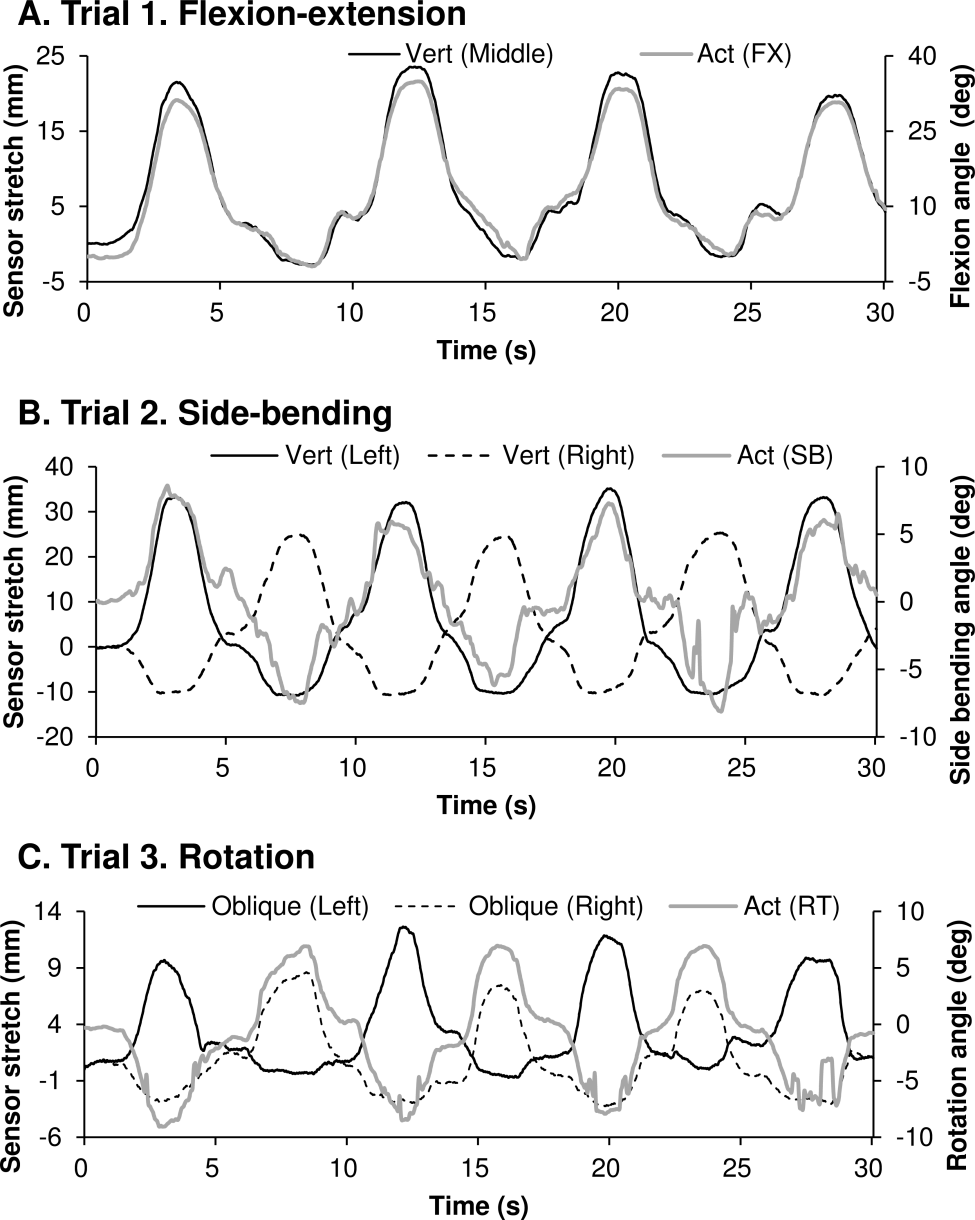
Typical time series of the angular displacement and stretch sensor outputs. Typical time series of the angular displacement of the flexion-extension (A), side-bending (B), rotation (C), and stretch sensor outputs in the three uniaxial movement trials. The gray straight lines represent the angular displacement of the lumbar joint. The black straight and dotted lines represent the stretch sensor outputs. The straight and dotted lines in panels (B) and (C) are in an anti-phase relationship with each other.

Fig 2 shows the displacements of sensor length and lumbar angles in flexion-extension (A), side-bending (B), and rotation movements (C). The vertical sensors were attached along the rotational direction of lumbar flexion-extension. Hence, these sensors exhibited a good correlation with the flexion-extension angle (Fig 2A). The vertical sensors were alternatively stretched in the right and left directions during the side-bending movement (Fig 2B). The oblique sensors stretched alternately in accordance with the rotation angle (Fig 2C).

**Fig 3:**
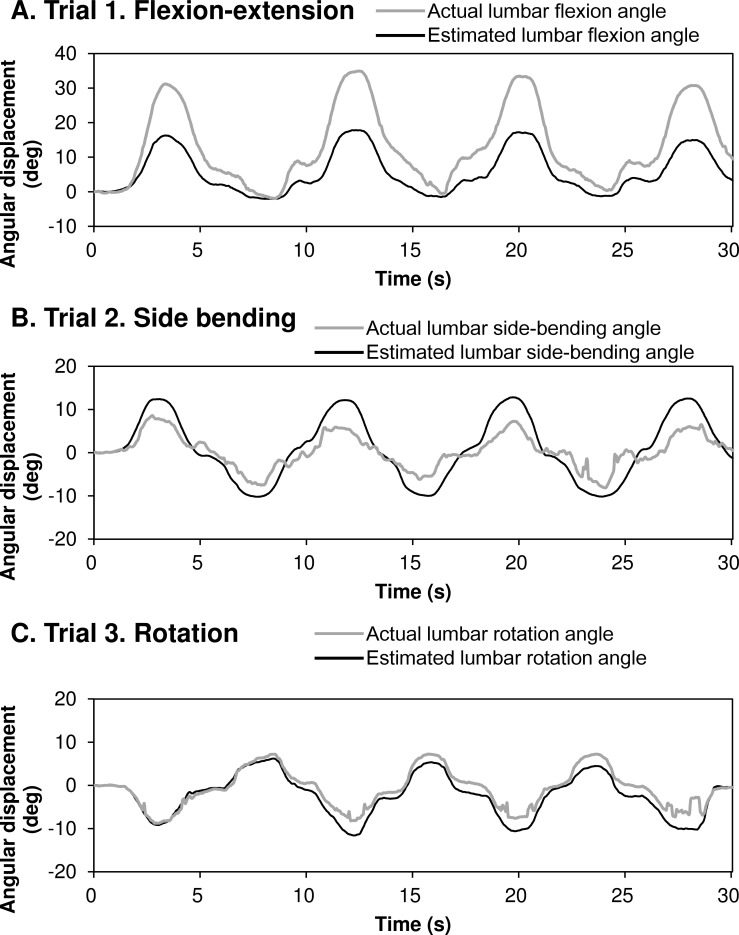
Representative angular displacements time series in uniaxial movements. Representative angular displacements for the (A) flexion-extension, (B) side-bending, and (C) rotation angles for the three uniaxial movement trials. The gray straight lines represent the angular displacement obtained from the optical motion capture system. The black straight lines represent the angular displacement estimated from the stretch sensor outputs.

We conducted a simple regression analysis to estimate the lumbar movements using the stretch sensor outputs. Fig 3 shows the actual and estimated lumbar movement angles for flexion-extension in trial 1 (Fig 3A), side-bending in trial 2 (Fig 3B), and rotation in trial 3 (Fig 3C). These data were collected from the same set of participants shown in Fig 2.

**Fig 4:**
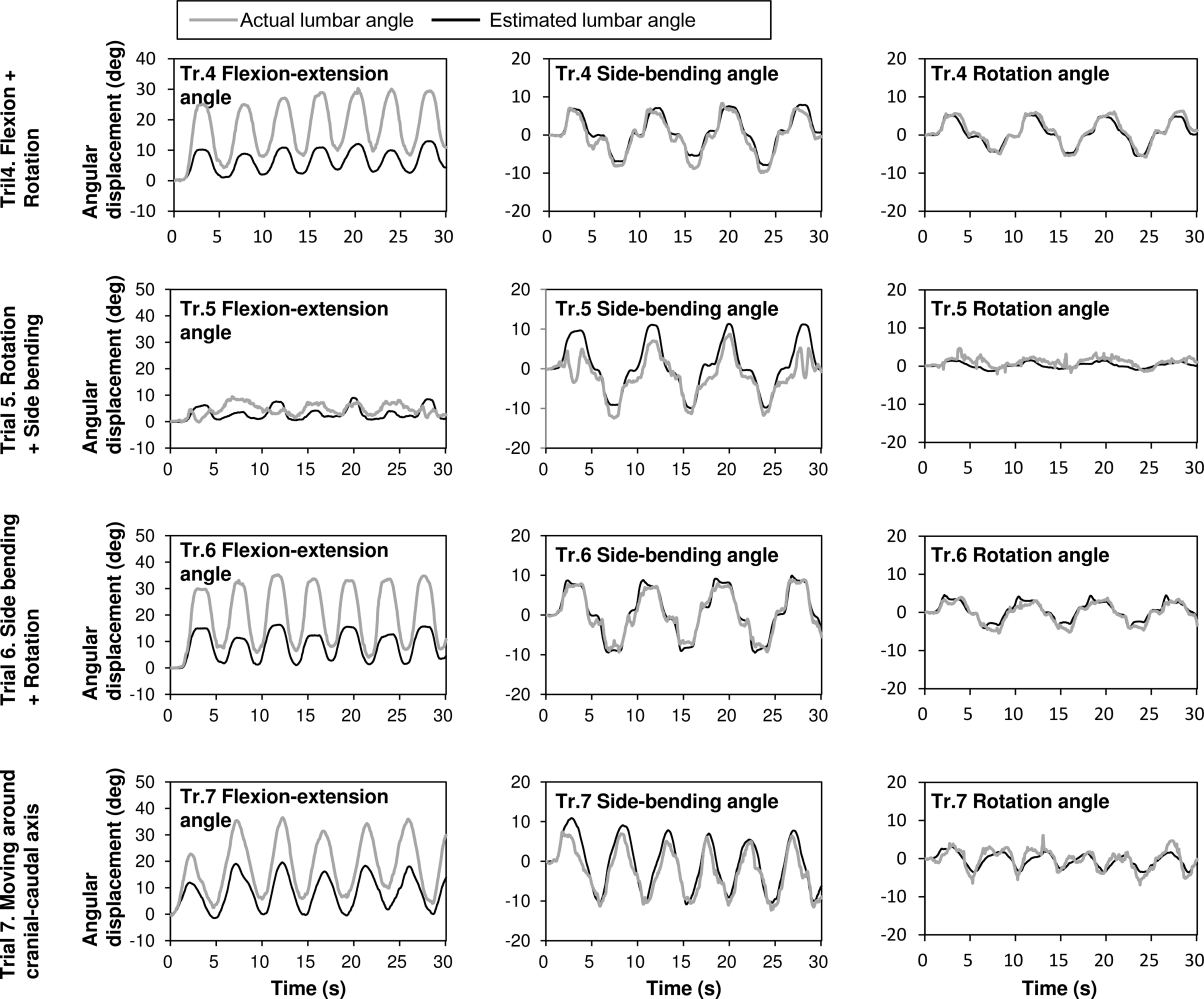
Typical time series of the actual and estimated angular displacements in multi-axial movements. Typical time series of the actual and estimated angular displacements obtained from optical motion capture (gray line) and stretch sensor (black line) when complex movements were performed in trials 4 to 7. From the top panel: angular displacements in the 4th experimental condition ((A) flexion and rotation), 5th experimental condition ((B) rotation and side-bending), 6th experimental condition ((C) flexion and side-bending), and 7th experimental condition ((D) turning trunk clockwise). The left panels represent flexion. The middle panels represent side-bending, while the right panels represent the rotation angles of the lumbar joint. The gray straight lines represent the actual angular displacement obtained from the optical motion capture system. The black straight lines represent the estimated angular displacement as calculated from the stretch sensor outputs.

Fig 4 illustrates the lumbar movement angles captured when performing the complicated movements in trials 4 to 7. The sensor outputs in all the trials were in phase with the actual lumbar movements. The amplitudes of the estimated angles appeared similar to the actual angles in side-bending and the rotation angles. The estimated angles as regards the flexion-extension angles were slightly smaller than the actual angles, especially for the large flexion-extension angle values.

The Pearson product correlation was calculated using the actual and estimated lumbar movements (Table 2). The average correlation coefficients for every trial were r = 0.68 (SD = 0.35, all *P* < 0.05), r = 0.60 (SD = 0.19, all *P* < 0.05), and r = 0.72 (SD = 0.18, all *P* < 0.05) for flexion-extension, side-bending, and rotation, respectively. Overall, these data indicated that the sensor outputs were either moderately or closely correlated to the actual lumbar movement angles. The correlation values were high for the trial, in which the range of motion was large. As regards flexion-extension angle, trials 1, 4, 6, and 7 showed r > 0.9 when the range of motion was over 18°. Meanwhile, trials 2, 3, and 5 exhibited an r-value < 0.5 for a range of motion below 10°.

**Table 2:** Correlation coefficients between actual and estimated lumbar angles.

Trial	Movement Condition	Flexion-extension		Side Bending		Rotation	
		Mean	SD^a^	Mean	SD^a^	Mean	SD^a^
1	Flexion	0.97	0.02	0.42	0.29	0.60	0.30
2	Side-bending	0.36	0.29	0.65	0.33	0.89	0.09
3	Rotation	0.17	0.36	0.29	0.48	0.94	0.06
4	Flexion + Rotation	0.97	0.01	0.89	0.14	0.80	0.24
5	Rotation + Side-bending	0.40	0.34	0.67	0.40	0.51	0.32
6	Side-bending + Flexion	0.91	0.07	0.69	0.41	0.78	0.22
7	Moving around cranial-caudal axis	0.94	0.03	0.58	0.32	0.52	0.56

^a^ SD: standard deviation

The estimation error was quantified using the RMS values between the actual and estimated angles. Table 1 shows the RMS values and the range of angles for each trial. The overall RMS errors were 2.35° (SD = 0.79), 1.98° (SD = 0.49), and 1.21° (SD = 0.50) for flexion-extension, side-bending, and rotation, respectively. The error ratios for the trial with a range of motion over two-thirds of the max ROM, which increased the risk of LBP [13,26], were 12.87% (SD = 3.54), 9.91% (SD = 2.72), and 10.19% (SD = 2.90) for flexion-extension, side-bending, and rotation, respectively. The error values in the trials with small correlation values were also small relative to those for the other trials.

**Fig 5:**
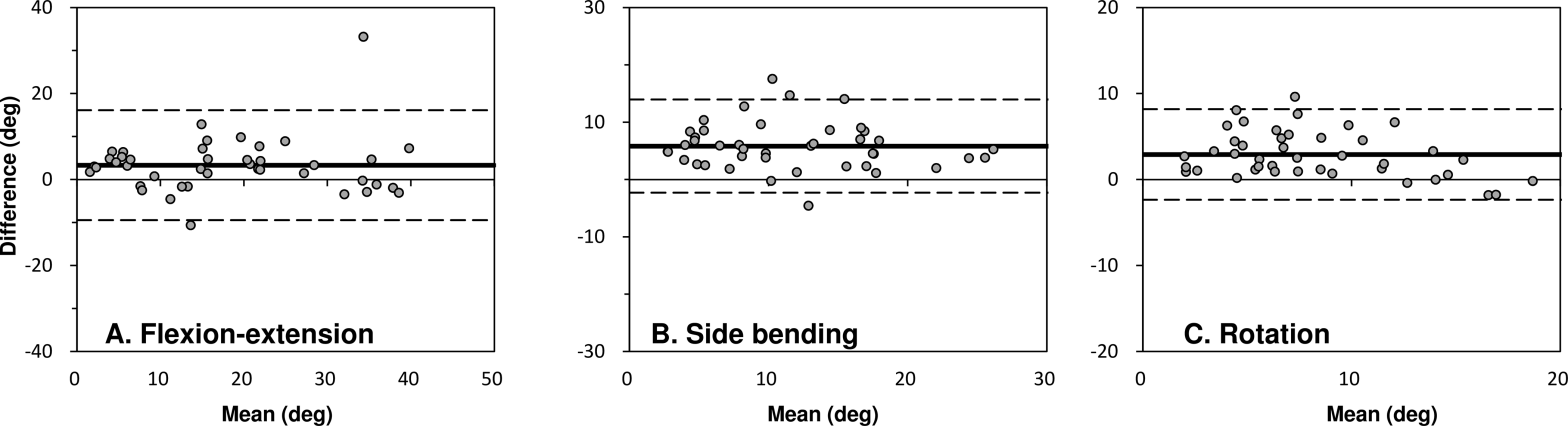
Bland–Altman plots of the evaluation results. The x-axis shows the average value of the angle obtained by stretch sensor and by the optical motion capture system. The y-axis shows the difference between the angles. The dashed lines denote ±1.96 SD (standard deviation), that 95% of the differences are in between the lines.

Differences of ROM-measurements were investigated using Bland-Altman analysis (Fig 5). The Bland-Altman plots demonstrated that differences in ROM-measurements are within the 1.96 SD with no outliers. The biases were 3.33°±6.52°, 5.83°±4.14°, and 2.91°±2.69° in flexion-extension, side-bending, and rotation, respectively. All regions in each direction between lower and upper limits included zero, indicating no systematic error.

## Discussion

We developed a new method to estimate lumbar angles in three dimensions using four body-fixed stretch sensors. The experimental evaluations showed that the sensor output signals were well correlated to the reference angles in all the three directions. The average of the residual errors between the actual and estimated angles was less than 3°. In addition, the mean differences in ROM-measurements were less than 6° and no systematic difference was observed.

The validity of the new sensor system was studied by comparing it with the optical motion capture system. The stretch sensor outputs showed a substantial correlation with the lumbar motion angles obtained by the optical motion capture system. Although lower back movements are an important factor for LBP occurrence, no clear consensus on the lumbar motion angles that induce LBP currently exists. Some studies suggested that over two-thirds of the ROM angles in the trunk bending task increased the LBP risk [13,26]. The experiment revealed strong correlation values (i.e., r > 0.9) for the trial in which the range of motion was large. The results suggested that the stretch sensor was suitable for monitoring large movements that increase the LBP risk.

As regards accuracy, the overall RMS errors were less than 3°. Previously, McGinley proposed that an error of 2° or less is considered acceptable in most common clinical situations [27]. Cuesta suggested that errors between 2° and 5° are likely to be regarded as reasonable with a careful interpretation of the measured data [28]. The data from this experiment indicated a reasonable accuracy for the measurements obtained by the stretch sensor system, but may require consideration when interpreting the results.

The error between the estimated and actual lumbar angles in this study was a result of two potential causes. The first cause is the crosstalk caused by skin stretching. The skin on the torso is like an elastic sheet covering the body. Joint motions in any direction stretch the skin. In fact, vertical sensors respond to both flexion-extension and side bending. The movements with a small range of motion showed relatively low correlation values with the low back angles measured by the optical motion capture, indicating that small movements are more susceptible to crosstalk. Further studies should aim to establish a sophisticated algorithm and a means of mounting the sensors to reduce the crosstalk effect. The second error factor is the decrease in the initial pre-strain (shortening length error). The sensor underestimates the angles if the sensor length is shortened. The development of a fixation methodology maintaining the initial length would further improve the measurement accuracy.

The benefits of the wearable motion capture system lie in its inherent portability and simplicity. Videos are capable of capturing objective lumbar angles even in occupational setting studies. However, the capture scope is limited to the area immediately in front of the camera. Multiple cameras are also required to obtain three-dimensional angles. Thus, previous studies only analyzed the movements on the sagittal plane [15,26]. In comparison with a camera-based system, the placement of the four stretch sensors used in the proposed method provided angular information from all the three directions. Moreover, the capture space was not limited. Recent longitudinal studies indicated that the duration of the task of bending forward from the trunk was not independently related to LBP. These studies also suggested that other motion angles, such as rotation, should be measured [29]. The proposed wearable system will be useful in future studies to objectively measure the tri-axial movements of workers such as healthcare workers who frequently move around as part of their duties.

An IMU sensor is another wearable system that can capture human movement. A number of studies reported the validity and reliability of inertial sensors during static and dynamic trunk movements aiming at clinical applications [28,30,31]. Previous studies reported that IMU sensors showed high accuracy in the lumbar trunk angle as compared to golden standard measurements, such as less than 5.8° RMS errors in the pitch axis. A recent study adapted this approach to measure the trunk flexion angles of blue-collar workers [20,29,32]. The computational problem is an important problem to obtain the angle data from IMU sensor. The angle data obtained from integration of angular velocity can be distorted by offset or other drifts during long-term monitoring. Recent studies investigated the validity of the lumbar angle estimation in the laboratory setting and proposed the algorism; accelerometer data is used together as a reference angle to correct drifts in gyroscope data, when velocity can be assumed small enough or near constant [30]. However, nurses and healthcare workers move irregularly depending on patients’ needs throughout their working time. To the best of our knowledge, the validity of long-term monitoring for the lumbar angle of healthcare workers during working time has not been investigated. Meanwhile, the stretch sensor signals can be translated to angle values using simple linear estimation without the need for integration. Further studies must be undertaken to compare the ability of these sensors in terms of accuracy as well as convenience to for use in long-term monitoring in healthcare workers.

There are four main limitations of this study. First, the participants were limited in that they were all healthy young adults. Second, even among the healthy population, the stretch of skin in the overweight population may be different from that in the normal weight population. Second, for clinical usage, practical calibration of the stretch sensor is required. Based on the high linearity between capacitance and squared sensing area, users need to measure at least two reference angles in each direction before capturing motion using the current stretch sensor system. For example, users measure the angle and sensor length in two postures such as an upright stance and standing with maximum trunk flexion. Development of a simple calibration protocol is important in applying the system to a clinical setting. Third, the results of this study did not provide any data on the validity of the stretch sensor measurements being representative of intersegmental lumbar spine kinematics, which is assumed to be related to LBP [33,34]. To demonstrate the validity of the approach, different study design and measurement methods, for example comparison with video fluoroscopy, would be required.

Wearable monitoring system can contribute to preventing LBP in respect to the following three points. 1) Real-time quantitative feedback of lower back movements is useful to teach appropriate movements to reduce lower back burdens in workplace training sessions. 2) An alert system can be created to notice the cumulative risk of LBP related to lower back movements. 3) It will be possible to capture objective evidence of workloads, which may motivate managers to change working environments.

Overall, the sheet stretch sensors attached to the back of young healthy adults provided lumbar angle information in three directions using a simple linear regression. The sensor had a lightweight, thin, and flexible nature. Hence, the wearable sensor system possessed great potential to monitor the lumbar angles of workers that move around as part of their duties.

## References

[1] Leboeuf-Yde C, Klougart N, Lauritzen T. How Common Is Low Back Pain in the Nordic Population? Spine. 1996;21(13):1518–25. doi: 10.1097/00007632-199607010-00005 8817778 10.1097/00007632-199607010-00005

[2] Deyo RA, Weinstein JN. Low back pain. The New England journal of medicine. 2001;344(5):363–70. Epub 2001/02/15. doi: 10.1056/NEJM200102013440508 . 11172169 10.1056/NEJM200102013440508

[3] Smedley J, Egger P, Cooper C, Coggon D. Manual handling activities and risk of low back pain in nurses. Occupational and environmental medicine. 1995;52(3):160–3. ; PubMed Central PMCID: PMCPMC1128180. 7735387 10.1136/oem.52.3.160 PMC1128180

[4] Niedhammer I, Lert F, Marne MJ. Back pain and associated factors in French nurses. International archives of occupational and environmental health. 1994;66(5):349–57. Epub 1994/01/01. . 7896421 10.1007/BF00378369

[5] Trinkoff AM, Lipscomb JA, Geiger-Brown J, Storr CL, Brady BA. Perceived physical demands and reported musculoskeletal problems in registered nurses. American journal of preventive medicine. 2003;24(3):270–5. Epub 2003/03/27. . 12657347 10.1016/s0749-3797(02)00639-6

[6] Videman T, Ojajarvi A, Riihimaki H, Troup JDG. Low back pain among nurses—A follow-up beginning at entry to the nursing school. Spine. 2005;30(20):2334–41. doi: 10.1097/01.brs.0000182107.14355.ca 16227898 10.1097/01.brs.0000182107.14355.ca

[7] Ono R, Yamazaki S, Takegami M, Suzukamo Y, Konno S, Kikuchi S, et al Patient-reported disability in the general Japanese population was associated with medical care visits for low back pain, regardless of pain intensity. J Orthop Sci. 2015;20(4):742–9. doi: 10.1007/s00776-015-0719-3 . 25862328 10.1007/s00776-015-0719-3

[8] Stewart WF, Ricci JA, Chee E, Morganstein D, Lipton R. Lost productive time and cost due to common pain conditions in the US workforce. JAMA. 2003;290(18):2443–54. doi: 10.1001/jama.290.18.2443 . 14612481 10.1001/jama.290.18.2443

[9] McDonald M, DiBonaventura M, Ullman S. Musculoskeletal pain in the workforce: the effects of back, arthritis, and fibromyalgia pain on quality of life and work productivity. J Occup Environ Med. 2011;53(7):765–70. doi: 10.1097/JOM.0b013e318222af81 . 21685799 10.1097/JOM.0b013e318222af81

[10] Hoogendoorn WE, Bongers PM, de Vet HC, Douwes M, Koes BW, Miedema MC, et al Flexion and rotation of the trunk and lifting at work are risk factors for low back pain: results of a prospective cohort study. Spine. 2000;25(23):3087–92. Epub 2001/01/06. . 11145822 10.1097/00007632-200012010-00018

[11] Leclerc A, Tubach F, Landre MF, Ozguler A. Personal and occupational predictors of sciatica in the GAZEL cohort. Occup Med (Lond). 2003;53(6):384–91. . 14514905 10.1093/occmed/kqg072

[12] Miranda H, Viikari-Juntura E, Punnett L, Riihimaki H. Occupational loading, health behavior and sleep disturbance as predictors of low-back pain. Scand J Work Environ Health. 2008;34(6):411–9. 19137202 10.5271/sjweh.1290

[13] Punnett L, Fine LJ, Keyserling WM, Herrin GD, Chaffin DB. Back disorders and nonneutral trunk postures of automobile assembly workers. Scand J Work Environ Health. 1991;17(5):337–46. . 1835131 10.5271/sjweh.1700

[14] Waters TR, Putz-Anderson V, Garg A, Fine LJ. Revised NIOSH equation for the design and evaluation of manual lifting tasks. Ergonomics. 1993;36(7):749–76. doi: 10.1080/00140139308967940 . 8339717 10.1080/00140139308967940

[15] Norman R, Wells R, Neumann P, Frank J, Shannon H, Kerr M. A comparison of peak vs cumulative physical work exposure risk factors for the reporting of low back pain in the automotive industry. Clinical biomechanics. 1998;13(8):561–73. . 11415835 10.1016/s0268-0033(98)00020-5

[16] Kilbom A. Assessment of physical exposure in relation to work-related musculoskeletal disorders—what information can be obtained from systematic observations? Scand J Work Environ Health. 1994;20 Spec No:30–45. . 7846490

[17] Hansson GA, Balogh I, Bystrom JU, Ohlsson K, Nordander C, Asterland P, et al Questionnaire versus direct technical measurements in assessing postures and movements of the head, upper back, arms and hands. Scand J Work Environ Health. 2001;27(1):30–40. . 11266144 10.5271/sjweh.584

[18] Balaguier R, Madeleine P, Rose-Dulcina K, Vuillerme N. Trunk kinematics and low back pain during pruning among vineyard workers-A field study at the Chateau Larose-Trintaudon. PloS one. 2017;12(4):e0175126 Epub 2017/04/07. doi: 10.1371/journal.pone.0175126 ; PubMed Central PMCID: PMCPMC5383154. 28384277 10.1371/journal.pone.0175126 PMC5383154

[19] Bolink SA, Naisas H, Senden R, Essers H, Heyligers IC, Meijer K, et al Validity of an inertial measurement unit to assess pelvic orientation angles during gait, sit-stand transfers and step-up transfers: Comparison with an optoelectronic motion capture system. Medical engineering & physics. 2016;38(3):225–31. doi: 10.1016/j.medengphy.2015.11.009 . 26711470 10.1016/j.medengphy.2015.11.009

[20] Villumsen M, Samani A, Jorgensen MB, Gupta N, Madeleine P, Holtermann A. Are forward bending of the trunk and low back pain associated among Danish blue-collar workers? A cross-sectional field study based on objective measures. Ergonomics. 2015;58(2):246–58. doi: 10.1080/00140139.2014.969783 . 25374330 10.1080/00140139.2014.969783

[21] Djuric-Jovicic MD, Jovicic NS, Popovic DB. Kinematics of gait: new method for angle estimation based on accelerometers. Sensors (Basel). 2011;11(11):10571–85. doi: 10.3390/s111110571 ; PubMed Central PMCID: PMCPMC3274301. 22346659 10.3390/s111110571 PMC3274301

[22] Nakamoto H, Ootaka H, Tada M, Hirata I, Kobayashi F, Kojima F. Stretchable Strain Sensor Based on Areal Change of Carbon Nanotube Electrode. Ieee Sens J. 2015;15(4):2212–8. doi: 10.1109/Jsen.2014.2377022

[23] Nakamoto H, Ootaka H, Tada M, Hirata I, Kobayashi F, Kojima F. Stretchable Strain Sensor With Anisotropy and Application for Joint Angle Measurement. Ieee Sens J. 2016;16(10):3572–9. doi: 10.1109/Jsen.2016.2535489

[24] Nakamoto H, Oida S, Ootaka H, Hirata I, Tada M, Kobayashi F, et al Design and Response Performance of Capacitance Meter for Stretchable Strain Sensor. Ieee Int C Int Robot. 2015:2348–53.

[25] Bland JM, Altman DG. Statistical Methods for Assessing Agreement between Two Methods of Clinical Measurement. Lancet. 1986;1(8476):307–10. 2868172

[26] Coenen P, Kingma I, Boot CR, Twisk JW, Bongers PM, van Dieen JH. Cumulative low back load at work as a risk factor of low back pain: a prospective cohort study. Journal of occupational rehabilitation. 2013;23(1):11–8. Epub 2012/06/22. doi: 10.1007/s10926-012-9375-z ; PubMed Central PMCID: PMCPMC3563950. 22718286 10.1007/s10926-012-9375-z PMC3563950

[27] McGinley JL, Baker R, Wolfe R, Morris ME. The reliability of three-dimensional kinematic gait measurements: a systematic review. Gait & posture. 2009;29(3):360–9. Epub 2008/11/18. doi: 10.1016/j.gaitpost.2008.09.003 . 19013070 10.1016/j.gaitpost.2008.09.003

[28] Cuesta-Vargas AI, Galan-Mercant A, Williams JM. The use of inertial sensors system for human motion analysis. Phys Ther Rev. 2010;15(6):462–73. doi: 10.1179/1743288X11Y.0000000006 ; PubMed Central PMCID: PMCPMC3566464. 23565045 10.1179/1743288X11Y.0000000006 PMC3566464

[29] Lagersted-Olsen J, Thomsen BL, Holtermann A, Sogaard K, Jorgensen MB. Does objectively measured daily duration of forward bending predict development and aggravation of low-back pain? A prospective study. Scand J Work Environ Health. 2016;42(6):528–37. Epub 2016/11/04. doi: 10.5271/sjweh.3591 . 27606607 10.5271/sjweh.3591

[30] Wong WY, Wong MS. Trunk posture monitoring with inertial sensors. Eur Spine J. 2008;17(5):743–53. Epub 2008/01/16. doi: 10.1007/s00586-008-0586-0 ; PubMed Central PMCID: PMCPMC2367407. 18196296 10.1007/s00586-008-0586-0 PMC2367407

[31] Bolink SAAN, Naisas H, Senden R, Essers H, Heyligers IC, Meijer K, et al Validity of an inertial measurement unit to assess pelvic orientation angles during gait, sit–stand transfers and step-up transfers: Comparison with an optoelectronic motion capture system*. Medical engineering & physics. 2016;38(3):225–31. http://dx.doi.org/10.1016/j.medengphy.2015.11.009. 26711470 10.1016/j.medengphy.2015.11.009

[32] Villumsen M, Holtermann A, Samani A, Madeleine P, Jorgensen MB. Social support modifies association between forward bending of the trunk and low-back pain: Cross-sectional field study of blue-collar workers. Scand J Work Environ Health. 2016;42(2):125–34. doi: 10.5271/sjweh.3549 . 26828769 10.5271/sjweh.3549

[33] McGregor A, Anderton L, Gedroyc W. The assessment of intersegmental motion and pelvic tilt in elite oarsmen. Medicine and science in sports and exercise. 2002;34(7):1143–9. Epub 2002/07/20. . 12131255 10.1097/00005768-200207000-00015

[34] Cheng JS, Carr CB, Wong C, Sharma A, Mahfouz MR, Komistek RD. Altered spinal motion in low back pain associated with lumbar strain and spondylosis. Evidence-based spine-care journal. 2013;4(1):6–12. Epub 2014/01/18. doi: 10.1055/s-0033-1341640 ; PubMed Central PMCID: PMCPMC3699246. 24436694 10.1055/s-0033-1341640 PMC3699246

